# Comparison of Pre-transfer Risk Assessment Tools Used for Critically Ill Patient Transport: A Retrospective Study

**DOI:** 10.7759/cureus.111512

**Published:** 2026-06-25

**Authors:** Aruvi Poomali, Ajay Ambalakkatte, Sreelal TP, Abhijit Mohanan, Nithyanand Madathil, Chippy Babu, Liza P Kadavana, Nizamudheen Kollanthodukayil

**Affiliations:** 1 Development Research, Indian Council of Medical Research, Nagpur, IND; 2 Trauma and Emergency, All India Institute of Medical Sciences, Nagpur, Nagpur, IND; 3 Community Medicine, Manipal University College Malaysia, Melaka, MYS; 4 Critical Care, Dr. Moopen's Medical College, Meppadi, IND; 5 Emergency Medicine Nursing, Ungoofaaru Regional Hospital, Raa Atoll, MDV; 6 Nursing, Bowden Derra Park Nursing Home, Cornwall, GBR; 7 Emergency Medicine Nursing, King Saud Medical City, Riyadh, SAU; 8 Emergency Medicine, National Ambulance, Sharjah, ARE

**Keywords:** critically ill patient transport, critically ill transport, emergency medicine, interhospital transport, prehospital care, triage

## Abstract

Objective

The objective of this study was to compare three scoring systems for pre-transfer risk assessment to enable the efficient utilization of transport infrastructure available while avoiding adverse events during interhospital patient transport.

Methods

This was a retrospective, cross-sectional study conducted in a tertiary care hospital situated in an aspirational district in Southern India. Trained personnel used three pre-transfer risk assessment tools to stratify the medical records of patients transported from the hospital during the study period. The data were analyzed to assess the sensitivity, specificity, and positive and negative likelihood ratios of the scoring systems.

Results

A total of 200 patient transports were included in the study. Cardiac emergency was the most common clinical diagnosis. A total of 35 patients had at least one major adverse event during transport. The Risk Score for Transport Patients (RSTP) classified 127 patients as high risk, the Transport Triage Tool (TTT) classified 133 as high risk, and the National Early Warning Score (NEWS2)-based tool classified 123 patients as high risk. Sensitivity and specificity of the RSTP scoring system to predict a major adverse event during transport were 77% and 39%, respectively. It also had a positive likelihood ratio of 1.27.

Conclusion

While the RSTP scoring system has the highest sensitivity and positive predictive value, further research is required to validate its performance in the Indian context.

## Introduction

Background

Interhospital transport is the movement of a patient between two medical centers. The development of highly specialized treatment options has led to a greater need for patient transfer between hospitals. Iwashyna describes the interhospital transfer system as an infrastructure that enables the smooth transport of patients to the hospital best suited to provide the care they need [1].

The transportation of a patient aims to ensure access to the best possible care. Treatment plans are often re-evaluated and modified according to the specialist care available at the receiving center. However reports suggest patient transportation may also be associated with disadvantages such as complications occurring during transportation, potential information loss at the receiving end, interruption in ongoing healthcare during the transfer out and back into the intensive care unit, delay in initiation of treatment at the receiving center, higher costs, and personal concerns like anxiety about the new environment and being away from family [1-3].

Orr et al. provide strong evidence that using specialized transport teams results in lower adverse events and increased survival, i.e., 23% (specialized) versus 9% (non-specialized), during interhospital transport [4]. Transport tools, checklists, and other documents help keep records and adhere to the mandatory protocols to ensure a smooth transition of patients from one environment to another, maintaining physiological and clinical stability [5]. Risk stratification systems for transporting patients aim to minimize the number of personnel involved in transfers.

In this context, it was imperative for the authors to identify a pragmatic and reliable scoring system for pre-transfer risk assessment to efficiently utilize the transport infrastructure available while avoiding adverse events during transport. Various risk indices for transport have been evaluated, but none have gained widespread acceptance.

The objective of this study was to compare the performance of the Risk Score for Transport Patients (RSTP), Transport Triage Tool (TTT), and National Early Warning Score (NEWS2)-based scoring system in predicting patients most likely to develop major complications during interhospital transport and thus deciding on the most appropriate team for transport [6-8]. Assessment of a higher risk would require a resident doctor to join the transport team. The primary outcome measure was the prognostic performance of these scoring systems described through relevant indices. The study is expected to guide the medical community working in similar geographical and socioeconomic conditions.

## Materials and methods

Study design and setting

This was a retrospective, cross-sectional study where personnel trained to use three scoring systems evaluated the paper-based medical records of patients transported out of the medical facility during a two-year period. The study was initiated after receiving approval from the Institutional Research Committee and the Institutional Ethics Committee (IEC/DMWIMS/Dec/2021-021). Informed consent was not required as it was a retrospective study. This study was conducted in a newly established 650-bedded tertiary care center with 30 critical care beds catering to six lakh people. The hospital is located in an aspirational district, Wayanad, in the Western Ghats of southern India [9]. Patients requiring services not available in the institution, after initial evaluation and stabilization, are transported to tertiary care hospitals located in a district center that is 80 kilometers away. The mean transport time to these centers is 120 minutes. Most of the transfers occur outside regular working hours, when specialist transport teams are unavailable.

Selection of the sample

We included all critically ill patients transported out of the hospital to tertiary care centers between 1 January 2020 and 31 December 2021. Those patients who were transported out of different intensive care units and the emergency medicine department, who were undergoing mechanical ventilation or advanced airway, required vasoactive drugs or were suspected to develop hemodynamic compromise during transport, including arrhythmias and shock, were considered as critically ill patients for this study. The case files of all patients included in the study were accessed through the medical records department of the hospital. The records were assessed for inclusion into the study using the following criteria: transported in advanced care ambulance, age more than 18 years and transport team comprising a doctor, and two emergency medicine technicians in the transport team. All case files with inadequate data were excluded.

Scoring systems

The risk stratification tools used in this study were the RSTP, the TTT, and the NEWS2 [6-8]. The RSTP score was developed in Spain and validated in Portugal. As per this scoring system, a score of more than 6 requires the patient to be accompanied by a doctor [6]. The TTT is a triaging tool developed and validated in the USA, where a cut-off of more than 0.43 requires the patient to be accompanied by a trained physician during transport [7,10]. The NEWS2 score-based clinical decision tool classifies the patient into low, medium, and high risk, with all except low-risk patients requiring a physician to accompany during transportation [8]. The tools are available in the Appendices.

Assessors

A senior emergency medicine technician (with a nursing degree, a certificate in prehospital care, and has been involved in critically ill patient transport for more than two years) and an emergency medicine technician trainee (with a nursing degree and pursuing a certificate course in prehospital care at the time of the study) were trained to use the three risk stratification tools using a 30-minute power point presentation and a table of exercises practiced over a period of one week at the start of the study.

Measurements

The demographic characteristics of the patients who were transported were collected. The clinical condition of the patient prior to transport, as recorded in the case file, was used to calculate the three risk assessment tools.

Each case sheet was assessed by the senior emergency medicine technician and the emergency medicine technician trainee separately, and the risks were calculated independently. Inter-rater reliability was calculated. All adverse events that occurred during transport were also collected during the medical record review. Major adverse events assessed included cardiac or respiratory arrest, a decrease of three points in Glasgow Coma Scale (GCS) score, a fall in the fraction of inspired oxygen (FiO2) ratio by 10, hypertension (systolic blood pressure (SBP) >200 mm Hg), hypotension (SBP <90 mm Hg), life-threatening arrhythmia, or need for any invasive procedure en route.

Outcomes

The primary outcome was the prognostic performance of these scoring systems assessed by their sensitivity, specificity, and positive and negative likelihood ratios.

Analysis

Following data collection, the patients were divided into two groups, high-risk and not high-risk, based on the three scoring systems. Descriptive statistics, including demographic profile, were expressed as mean and standard deviation. The discrimination of the RSTP and TTT was tested using the receiver operating characteristic (ROC) curve and area under the curve (AUC). The rule of 100 for a simple comparison of two groups, which states that a sample size of approximately 100 per group is often sufficient to detect a moderate effect size with reasonable power, was used. Data were analyzed using Microsoft Excel (Microsoft Corporation, Redmond, WA) and R program version 4.5.1 (R Foundation for Statistical Computing, Vienna, Austria).

## Results

Characteristics of study subjects

A total of 350 patient transport events occurred during the study period. Of these 350 transports, 200 fulfilled the inclusion and exclusion criteria for the study. This included 147 male and 53 female patients. The average age of the patients transported was 53.8 years. The most common clinical diagnosis of transported patients was cardiac emergency. A total of 35 patients had at least one major adverse event during transport. Three patients had cardiac arrest. A total of 65 patients required vasopressors, and 92 required mechanical ventilation during transport. There was no mortality during transport. The clinical diagnoses, along with the nature of adverse events that were encountered during transport, are detailed in Table 1.

**Table 1:** Baseline characteristics. GCS: Glasgow Coma Scale; SpO2: oxygen saturation; SBP: systolic blood pressure; RSTP: Risk Score for Transport Patients; TTT: Transport Triage Tool; NEWS2: National Early Warning Score.

Gender	Number	Percentage
Male	147	73.5
Female	53	26.5
Age	Mean	Standard deviation
Total sample	53.86	17.04
Clinical diagnosis of transported patients		
Diagnosis	Number	Percentage
Traumatic brain injury	21	10.5
Chest injury	4	2
Acute coronary syndrome	34	17
Intracranial hemorrhage	18	9
Traumatic spinal cord injury	6	3
Upper gastrointestinal hemorrhage	10	5
Toxicology	9	4.5
Polytrauma	12	6
Pregnancy complications	4	2
Severe sepsis	15	7.5
Respiratory failure	27	13.5
Multiple organ dysfunction syndrome	7	3.5
Ischemic stroke	14	7
Heart failure	8	4
Electrical injury	2	1
Encephalopathy	3	1.5
Guillain-Barré syndrome	2	1
Seizure	2	1
Bowel obstruction	1	0.5
Burns	1	0.5
Major adverse event in transported patients		
Adverse events	Number	Percentage
Cardiac arrest	3	1.5
Fall in GCS by more than 3 points	2	1
Decrease in SpO2 by more than 5%	4	2
Hypertension during transport (SBP > 200)	5	2.5
Hypotension during transport (SBP < 90)	17	8.5
Any invasive procedure during transport	1	0.5
Airway obstruction	2	1
New arrhythmia during transport	2	1
Pneumothorax	2	1
Convulsion during transport	1	0.5
Accidental extubation	1	0.5
Agitation	7	3.5
New-onset ECG changes suggestive of acute coronary syndrome	4	2
Scores	Mean	Standard deviation
RSTP	7.4	2.57
TTT	1.21	0.83
NEWS2 score	4.93	2.78

Main results

The RSTP score classified 127 patients as high risk with a score > 6, 69 patients as low risk with a score of 3 to 6, and four patients as low risk with a score < 3. The TTT classified 133 patients as high risk, 60 patients as moderate risk, and seven patients as low risk. The NEWS2-based tool classified 123 patients as high risk, 42 patients as moderate risk, and 35 as low risk. Table 2 presents the distribution of low-risk and high-risk groups across all three scoring systems. The average RSTP score was 7.4, the average TTT score was 1.21, and the average NEWS2 score was 4.93.

**Table 2:** Distribution of high-risk and not high-risk patient transports across three different scoring systems. RSTP: Risk Score for Transport Patients; TTT: Transport Triage Tool; NEWS2: National Early Warning Score.

Risk assessment tool	High risk	Not high risk
RSTP	127 (63.5%)	73 (36.5%)
TTT	133 (66.5%)	67 (33.5%)
NEWS2-based pre-transfer risk assessment	123 (61.5%)	77 (38.5%)

Of the patients classified as non-high risk as per the RSTP score, there were eight patients with major adverse events: one had cardiac arrest, two patients had agitation, two patients developed a fall in oxygen saturation, one patient developed hypertension, and two patients developed ECG changes suggestive of new acute coronary syndrome (ACS). Of the patients classified as non-high risk by TTT, nine patients had major adverse events, one had cardiac arrest, one had agitation, two had a fall in oxygen saturation, one had hypertension, one had pneumothorax, one had hypotension, and two had ECG changes suggestive of new ACS. Of the patients classified as low risk by the NEWS2-based scoring system, nine patients had major adverse events, one had cardiac arrest, two had hypotension, two had agitation, two had new-onset ECG changes suggestive of ACS, and two had a fall in oxygen saturation. The patient who had a cardiac arrest was classified as non-high risk by all three systems.

Sensitivity and specificity of the RSTP scoring system to predict major adverse events during transport were 77% and 39%, those of the TTT were 74% and 35%, and those of the NEWS2-based scoring system were 74% and 41%, respectively. RSTP had the highest positive likelihood ratio of 1.27 compared to 1.26 for NEWS2-based pre-transfer risk assessment and 1.15 for TTT. Table 3 depicts the comparison of the three scoring systems.

**Table 3:** Performance characteristics of three scoring systems. RSTP: Risk Score for Transport Patients; TTT: Transport Triage Tool; NEWS2: National Early Warning Score.

	TTT		RSTP		NEWS2-based pre-transfer risk assessment	
	Value	CI	Value	CI	Value	CI
Sensitivity	74.29%	56.74% to 87.51%	77.14%	59.86% to 89.58%	74.29%	56.74% to 87.51%
Specificity	35.15%	27.89% to 42.96%	39.39%	31.89% to 47.29%	41.21%	33.62% to 49.13%
Positive likelihood ratio	1.15	0.91 to 1.43	1.27	1.02 to 1.58	1.26	1.00 to 1.60
Negative likelihood ratio	0.73	0.40 to 1.33	0.58	0.31 to 1.10	0.62	0.35 to 1.13

**Figure 1:**
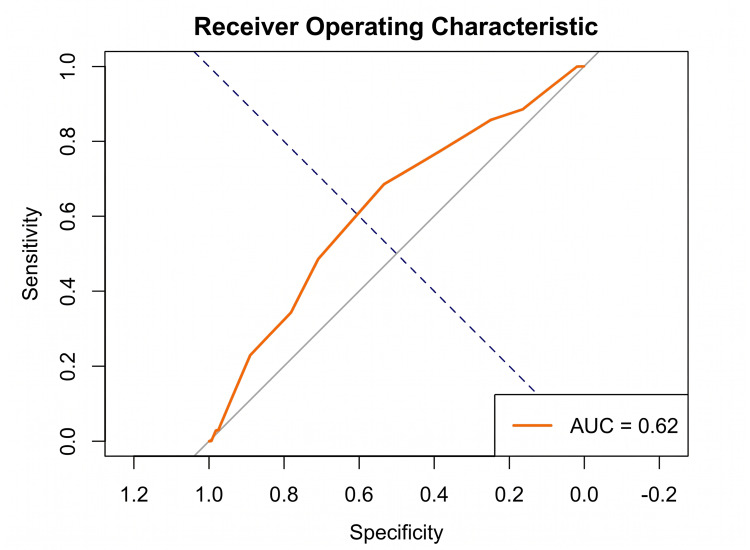
ROC curve for RSTP. ROC: receiver operating characteristic; AUC: area under the curve; RSTP: Risk Score for Transport Patients.

**Figure 2:**
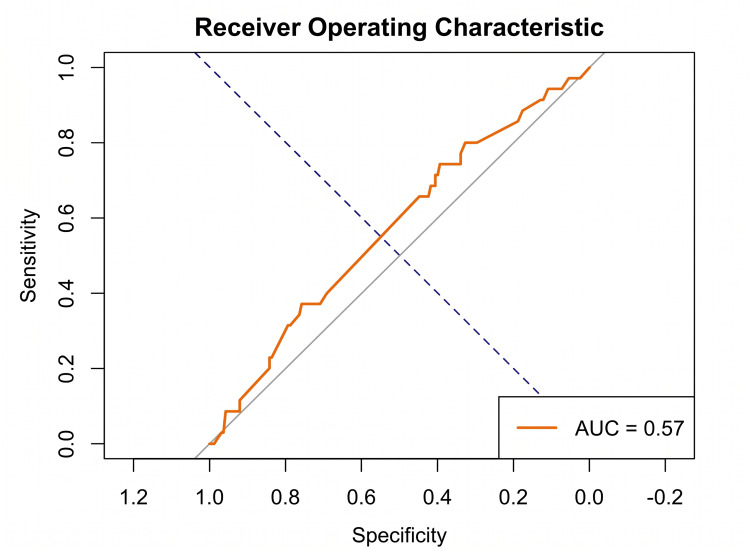
ROC curve for TTT. ROC: receiver operating characteristic; AUC: area under the curve; TTT: Transport Triage Tool.

The ROC curve for RSTP is shown in Figure 1. The discrimination power of RSTP AUC showed acceptable discrimination (AUC: 0.624; standard error (SE): 0.052; p = 0.021; 95% CI: 0.522 to 0.727). The ROC curve for TTT is shown in Figure 2. The discrimination power of TTT AUC was poor (AUC: 0.572; SE: 0.053; p = 0.181; 95% CI: 0.469 to 0.676).

Inter-rater reliability of the three scoring systems was calculated individually. The kappa coefficients were 0.36 for RSTP, 0.28 for TTT, and 0.43 for the NEWS2-based system. All the scoring systems showed only fair agreement as per the kappa statistic. The intra-item and intra-rater reliability were not explored.

## Discussion

Interhospital transport from our center to specialist centers during the study period was conducted safely, with no mortality reported during the transport phase. The most common clinical diagnoses of transported patients were cardiac emergencies and trauma-related emergencies. Multiple studies report improved patient outcomes following interhospital transfer to specialist centers in patients with these diagnoses [11-13]. Shock was the most common major adverse event, likely attributable to the high proportion of patients with severe sepsis, cardiac emergencies, or trauma who were already receiving vasopressors prior to transport.

The transport team for interhospital transport in our hospital consisted of a senior emergency medicine technician, an emergency medicine technician trainee, and a junior resident. This is similar to the reports of a study by Ong et al., where the authors reviewed the differences between emergency medical services in Asian countries and the Western world [14].

In this study, we compared the effectiveness of three risk stratification tools (RSTP, TTT, and NEWS2) in classifying patients as high risk or not high risk. This is the first time such tools have been studied in the Indian context. Our results showed that RSTP had the highest sensitivity and positive likelihood ratio, while the NEWS2-based pre-transfer risk assessment tool had the highest specificity.

TTT had low sensitivity, specificity, and positive likelihood ratio among the three tools studied. This contrasts with the findings of Swickard et al. in the United States, who reported higher sensitivity and specificity [10]. This discrepancy could be due to the significant difference in the experience of data collectors; Swickard et al.’s study had an average of 16 years of experience in critical care transport, compared to two years in our study [10]. The subjective nature of TTT may require more experienced assessors.

In a study by Markakis et al., RSTP showed a higher specificity of 65% and a sensitivity of 78% [15]. Swickard et al. evaluated the number of interventions required beyond the transport team’s scope of practice and found that the RSTP score had a sensitivity of 71% and a specificity of 73% [10]. The sensitivity obtained in both these studies is comparable to that of the current study. The lower specificity in our study could be because all adverse events during transportation were considered, regardless of whether they were managed by the transport team.

The NEWS2-based pre-transfer risk assessment tool was adapted from the West Yorkshire Critical Care Operational Delivery Network under the NHS [8,16]. There was no previous data available regarding the sensitivity and specificity of this tool.

The discriminatory power, as evaluated by the AUC of the ROC, for TTT and RSTP in our study was 0.572 and 0.624, respectively, which were lower than the values reported by Swickard et al. (0.81 and 0.74, respectively). Swickard et al. also found that the inter-rater reliability had a Cohen’s kappa value of 0.574, while our study reported a value of 0.21 [10]. This decrease could be attributed to the level of experience of the providers in critical care transport. Inter-rater reliability was higher for RSTP and the NEWS2-based pre-transfer risk assessment tool compared to TTT, likely due to the more objective nature and ease of use of these scoring systems. RSTP included components with binary answers, and NEWS2 was already in use in the hospital as part of the rapid response team (RRT), contributing to its ease of use.

While RSTP appears to be a promising tool for risk stratification, further research is needed to validate its performance and ensure it is an optimal tool for different clinical settings and patient populations. We believe this study will be of particular interest to low- and middle-income countries with medical personnel working in similar geographical and socioeconomic backgrounds to ours. Future studies should aim to validate the findings of this study in larger, prospective cohorts and involve more experienced caregivers to improve the generalizability of the results.

Limitations

Several limitations of this study should be acknowledged. The retrospective design and reliance on medical records may have introduced bias. A prospective design and a larger sample size would have enabled us to generalize the findings. The low experience level of the transport team, compared to international standards, may have influenced the accuracy of risk stratification and the overall outcomes of the study.

## Conclusions

This study provides valuable insights into the comparative effectiveness of three risk stratification tools in the context of interhospital transport of critically ill patients in India. This study confirms the feasibility of using all three scoring systems in routine clinical settings. Further research is needed to validate the performance of RSTP in different clinical settings and patient populations. A tool that will objectively match the available infrastructure with the risk involved in patient transport will enable efficient utilization of resources by medical personnel working in resource-limited settings.

## Appendices

The scoring systems that were used for risk stratification in the study are presented in the table below.

**Table 4:** Risk stratification tools used in the study. TTT: Transport Triage Tool; NEWS2: National Early Warning Score; SpO2: oxygen saturation; AMI: acute myocardial infarction; GCS: Glasgow Coma Scale; FiO2: fraction of inspired oxygen.

Risk Score for Transport Patients							
Measurement category		Clinical criteria/Status					Score
1. Hemodynamic		Stable					0
		Moderately stable (requires volume < 15 ml/min in adults)					1
		Unstable (requires volume > 15 ml/min or inotropics or blood)					2
2. Arrhythmias (existing/probable)		No					0
		Yes, not serious (and AMI after 48 hours)					1
		Serious (and AMI in the first 48 hours)					2
3. ECG monitoring		No					0
		Yes (desirable)					1
		Yes (essential)					2
4. Intravenous line		No					0
		Yes					1
		Pulmonary artery catheter					2
5. Provisional pacemaker		No					0
		Yes (non-invasive)					1
		Yes (endocavity)					2
6. Respiration		Respiratory rate between 10 and 14 breaths/min in adults					0
		Respiratory rate between 15-35 breaths/min in adults					1
		Apnea, < 10 or > 36 breaths/min, or irregular breathing					2
7. Airway		None required					0
		Yes (Guedel/Oropharyngeal tube)					1
		Yes (intubation or tracheostomy)					2
8. Respiratory support		No					0
		Yes (oxygen therapy)					1
		Yes (mechanical ventilation)					2
9. Assessment		GCS = 15					0
		GCS = 8–14					1
		GCS = <8 and/or neurological disorder					2
10. Techno pharmacological support (actual or en route)		None					0
		Group I: Inotropic agents, vasodilators, antiarrhythmics, bicarbonate, analgesics, antiepileptics, steroids, mannitol 20%, thrombolytic agents, naloxone, thoracic tube (chest tube), suction equipment					1
		Group II: Inotropics + vasodilators (concomitant), MAST (military anti-shock trousers), infant incubator, general anesthetics, uterine relaxants (tocolytics)					2
Classification based on the score: Low risk = 0 to 3; medium risk = 3 to 6; high risk ≥ 6				Classification used in the study: High risk ≥ 6; not high risk = 0 to 6			
Transport Triage Tool							
Category	Description						Score
Predictability	Transport course not predictable or predictably negative; uncertain, uncommon patient population/illness; not following a predictable clinical course. Note: All time-sensitive emergencies are rated 1.						1
	Moderately predictable; wavering; clinical course could go either direction; complications possible but not immediately expected; common process at critical time that may or may not follow a predictable course.						3
	Highly predictable; certain; common patient population/illness following a predictable course.						5
Stability	Minimally stable; labile; unresponsive to therapies, high risk of death; likely to require interventions during transport to maintain homeostasis (e.g., medication titrations, new medications, or ventilator titrations).						1
	Condition is changeable but would not be adversely affected by no interventions for intervals.						3
	Highly stable; responsive to therapies; low risk of death. Unlikely to need therapeutic intervention to maintain homeostasis during transport.						5
Complexity	Highly complex; intricate; vague; atypical presentation; multiple interacting technologies, therapies, and comorbidities.						1
	Moderately complex; moderately involved patient dynamics.						3
	Minimally complex; routine; clear-cut; typical presentation.						5
Resiliency	Minimally resilient; unable to mount a response; minimal reserves.						1
	Moderately resilient; able to mount a moderate response; able to mount some compensatory response.						3
	Highly resilient; able to mount and maintain a response; strong reserves.						5
Vulnerability	Highly vulnerable; susceptible; unprotected; fragile; vulnerability may be due to technology, chemistry, or pathology.						1
	Moderately vulnerable; somewhat susceptible; somewhat protected.						3
	Minimally vulnerable; safe; not fragile.						5
TTT score = [11/(3P+3S+2C+2V+R)]+P(1)+S(1), where P represents the predictability score; S, the stability score; C, the complexity score; V, the vulnerability score; and R, the resiliency score. P (1) is a predictability premium, and S (1) is a stability premium. Predictability premium adds 1 point to the total score if predictability is rated as 1 (low predictability), and the stability premium adds 1 point to the total score if stability is rated as 1 (low stability)							
Classification based on the score: High risk = >0.43; medium risk = 0.22 to 0.42; low risk = <0.21		Classification used in the study: High risk = >0.43; not high risk = 0 to 0.42					
NEWS2-based pre-transfer risk assessment							
Physiological parameter	Score: 3	Score: 2	Score: 1	Score: 0	Score: 1	Score: 2	Score: 3
Respiratory rate (bpm)	≤8		9-11	12-20		21-24	≥25
Scale 1: SpO_2_ (Standard)	≤91%	92-93%	94-95%	≥96%			
Scale 2: SpO_2_ (Hypercapnic)	≤83%	84-85%	86-87%	88-92% or ≥ 93% on room air	93-94% on oxygen	95-96% on oxygen	≥97% on oxygen
Air or supplemental oxygen		Supplemental oxygen		Room air			
Temperature (°C)	≤35.0		35.1-36.0	36.1-38.0	38.1-39.0	≥39.1	
Systolic blood pressure (mmHg)	≤90	91-100	101-110	111-219			≥220
Heart rate (bpm)	≤40		41-50	51-90	91-110	111-130	≥131
Consciousness				Alert			New confusion/Voice/Pain/Unresponsive
Risk level	Clinical parameters & scores						
Low risk	NEWS2: 1-4. Airway: Maintaining own airway. Oxygenation: FiO_2_ < 0.4. Metabolic: Base deficit 0 to –4 mmol/L. Hemodynamic: Not requiring inotrope or vasopressor support. Neurology: GCS 14. Temperature: Normothermic						
Medium risk	NEWS2: 5-6. Airway: Maintaining own airway. Oxygenation: FiO_2_ < 0.6. Metabolic: Base deficit -4 to -8 mmol/l. Hemodynamic: Requiring low-dose inotrope or vasopressor support (<0.2 mg/kg/min^-1^). Neurology: GCS: 9-13. Temperature: Mild hypothermia or hyperthermia						
High risk	NEWS2: 7 or more. Airway: Intubated/ventilated. Oxygenation: FiO_2_ > 0.6. Metabolic: Base deficit worse than -8 mmol/l. Hemodynamic: Cardiovascularly unstable and/or requiring inotrope or vasopressor support (>0.2 mg/kg/min^-1^). Temperature: Moderate hypothermia or hyperthermia. Injury profile: Major trauma (e.g., head injury, chest, abdominal, or pelvic injury)						
Classification used in the study: High risk was considered as high risk itself, and low and medium risk were considered not high risk.							
